# Identification of a novel EXT1 mutation in patients with hereditary multiple exostosis by exome sequencing

**DOI:** 10.3892/or.2014.3610

**Published:** 2014-11-21

**Authors:** HONGJIE LIU, SONG WU, LI DUAN, WEIMING ZHU, SHIQUAN ZHANG, XIAOXIAO HU, WENLONG JIA, GUOSHENG YANG, CHUNXIAO LIU, WEIPING LI, LEI YANG, LIJUN GUO, YOUCHENG LIN, YONGQIANG WANG, MEIJIAN HE, ZHAO YANG, YINGYING HE, ZHIMING CAI, DAPING WANG

**Affiliations:** 1 Shenzhen Second People’s Hospital, The First Affiliated Hospital of Shenzhen University, Shenzhen 518000, P.R. China; 2 Zhongshan School of Medicine, Sun Yat-Sen University, Guangzhou 510000, P.R. China; 3 College of Life Science, University of Chinese Academy of Sciences College, Beijing 100049, P.R. China; 4 BGI Education Centre, University of Chinese Academy of Sciences, Beishan Industrial Zone, Shenzhen 518083, P.R. China; 5 BGI Tech Solutions Co., Ltd., Beishan Industrial Zone, Shenzhen 518083, P.R. China; 6 Key Laboratory of Infection and Immunity of CAS, Institute of Biophysics, Chinese Academy of Sciences, Beijing 100101, P.R. China; 7 Anhui Medical University, Hefei 230000, P.R. China; 8 Department of Urology, Zhujiang Hospital of Southern Medical University, Guangzhou 510000, P.R. China; 9 University of Chinese Academy of Sciences, Beijing 100049, P.R. China

**Keywords:** hereditary multiple exostosis, Ext1, exome sequencing, immunohistochemistry, truncated protein

## Abstract

Hereditary multiple exostosis (HME) is an autosomal inherited skeletal disease whose etiology is not fully understood. To further understand the genetic spectrum of the disease, we analyzed a five-generation Chinese family with HME that have observable inheritance. Exome sequencing was performed on three HME individuals and three unaffected individuals from the family. A downstream study confirmed a new C deletion at codon 442 on exon 5 of the exostosin-1 (EXT1) gene as the only pathogenic site which generated a stop codon and caused the truncation of the protein. We rediscovered the deletion in other affected individuals but not in the unaffected individuals from the family. Upon immunohistochemistry assay, we found that the EXT1 protein level of the patients with the novel mutation in our study was less than the level in the patients without the EXT1 mutation from another unrelated family. For a deeper understanding, we analyzed the mutation spectrum of the EXT1 gene. The present study should facilitate a further understanding of HME.

## Introduction

Hereditary multiple exostosis (HME) is an autosomal dominant disorder with an incidence of 1 in 50,000 (1). Thus, it is one of the most common inherited musculoskeletal disorders. Approximately 20% of reported cases have no family history of multiple exostosis (2). There are 3 features of HME. First, patients show multiple benign exostoses, typically located at the juxta-epiphyseal region of bones. Second, it has an obvious hereditary property. Third, and the most severe complication, is the characteristic of malignant transformation (3).

Previous studies have reported that the EXT family is responsible for HME. The EXT family was reported to encode proteins linked to the biosynthesis of heparan sulfate (HS) (4). HS is an essential molecule and dysfunction could cause HME. The EXT family consists of exostosin-1 (EXT1) (5), exostosin-2 (EXT2) (6), exostosin-3 (EXT3) (7), exostosin-like 1 (EXTL1) (8), exostosin-like 2 (EXTL2) (9) and exostosin-like 3 (EXTL3) (10). Among these, EXT1 has been suggested to be responsible to a large extent for HME (9,11).

With the rapid development of DNA sequencing technology, the power of exome sequencing for gene mapping of diseases has been demonstrated (12–14). Exome sequencing can help to identify novel causal genetic variants, as it covers all exon regions.

In the present study, we performed exome sequencing on 3 affected and 3 unaffected individuals from a HME family. After filtering out the mutations from the dbSNP database (build 132), the candidate mutations were validated on all of the patients from the HME family by Sanger sequencing. Further validation was also performed on three members from another unrelated HME family. Additionally, immunohistochemisty was conducted to provide a deeper insight into the importance of the identified causal gene mutation. Furthermore, the mutation spectrum of the EXT1 gene was analyzed.

## Materials and methods

### Subjects

**Figure 1:**
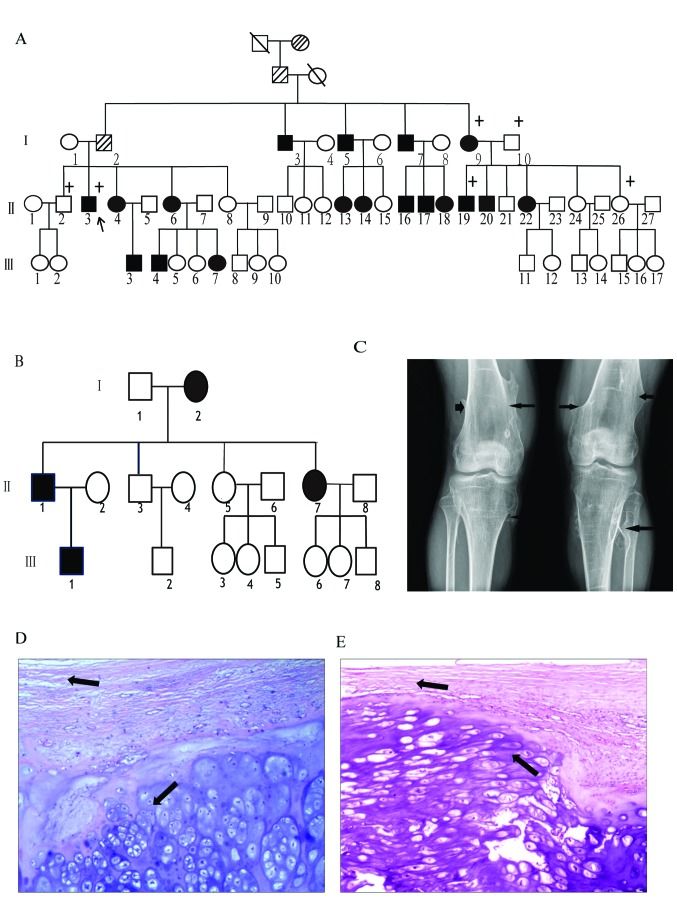
Pedigree structure and the clinical phenotype of the family enrolled for the exome sequencing analysis. (A) The genealogical tree of the family with hereditary multiple exostosis (HME) in the study. ‘+’ indicates those individuals examined and sequenced. The patient indicated by the arrow represents the proband. Black boxes or ellipses represent living patients, the white boxes or ellipses represent the healthy individuals, the boxes or ellipses filled with hashed lines represent deceased patients and the boxes or ellipses interrupted by a line segment represent deceased healthy subjects. (B) Another unrelated HME family (I1, I2 and II7). (C) Typical lesions of HME in the proband. (D and E) Toluidine blue (TB) and hematoxylin and eosin (H&E) staining of the proband. The upward arrows indicate the perichondrium and the downward arrow indicates the cartilage cap.

For exome sequencing, we selected 3 affected cases and 3 unaffected individuals (Table I) from a five-generation HME family (Fig. 1A) from the Henan Province of China. Sixteen affected cases, 13 unaffected individuals in the family and 3 individuals (2 affected cases and 1 unaffected) from another unrelated family (Fig. 1B) were selected for further validation. All of the participants were informed and consented with the specimen collection.

After filling in the informed consent form, all of the individuals were carefully examined by at least 2 experienced doctors. All of the cases were diagnosed as HME, while all of the unaffected individuals were healthy.

Venous blood samples were collected from all members who participated. Genomic DNA was extracted from peripheral blood by standard procedures for exome sequencing. In this study, all steps were conducted according to the Declaration of Helsinki Principles. The use of human subjects in this report was approved by the Shenzhen Second People’s Hospital Institutional Review Committee.

### Exome capture and sequencing

Exome capture was performed with the Human All Exon V3 (Agilent Technologies, Santa Clara, CA, USA) and the sequencing was conducted with the HiSeq 2000 platform (Illumina Inc., San Diego, CA, USA). Genomic DNA samples were randomly fragmented for library conducted and base-pair peaks of 150–200 bp were selected and then adapters were ligated to both ends of the fragments. The adapter-ligated templates were purified by Agencourt AMPure SPRI beads, and fragments with insert size ~200 bp were excised. Extracted DNA was amplified by ligation-mediated polymerase chain reaction (LM-PCR), purified and hybridized to the SureSelect Biotinylated RNA Library (BAITS) for enrichment. Hybridized fragments were bound to the strepavidin beads whereas non-hybridized fragments were washed out after 24 h. The captured LM-PCR product was subjected to the Agilent 2100 Bioanalyzer to estimate the magnitude of enrichment. All of the steps were performed according to the manufacturer’s recommendations. Each captured library was then loaded onto the Hiseq 2000 platform and sequencing was performed with read lengths of 90 bp, which provided at least a 50× average depth for each sample.

### Read mapping and variant detection

Reads with adapters, the N’s percent >10% and reads whose low quality bases (quality value ≤5) were >50% were filtered. The clean reads of each individual were aligned to the human reference genome (NCBI Build 36.3, hg19) using Burrows-Wheeler transform (BWA; Cambridge, UK); the parameter were set as ‘-o 1 -e 50 -m 100,000 -t 4 -i 15 -q 10’. Reads that had duplicated aligned sites were removed; the remaining reads mapped on or near the target were collected for subsequent analyses and variant calling.

For the SNP calling, we used Short Oligonucleotide Analysis Package (SOAPsnp, China) (15). A consensus genotype with Phred-like quality of at least 20 and at least 4× coverage depth was considered to be a high-confidence genotype. The genotypes that were different from the reference were extracted as candidate SNP and the SNP list was filtered with the followed criterion: Phred-like SNP quality ≥20, overall depth of 4 to 1,000×.

Genome Analysis Toolkit (GATK; Broad Institute, Cambridge, MA, USA) (16) was used to call InDel and the results were filtered as follows: After gap aligning the sequence reads with default parameters to the human reference (hg19), local realignment of the BWA-aligned reads using the GATK IndelRealigner were performed and the final InDels were called by GATK IndelGentotyperV2 according to the recommendations of the software.

### Functional annotation of genetic variants

With ANNOVAR (USA) (17) the variants were annotated and categorized into missense, nonsense, synonymous, splice-site and insertion/deletion mutations. After filtering out synonymous and nonframeshift, we obtained mutations which were likely to be deleterious and then filtered against Chinese Han SNP data available in the dbSNP database (build 132). We chose those variants that were shared by all affected but not any unaffected individual as candidate casual variants.

### Sanger sequencing

Sanger sequencing was performed to confirm the identified variants found by exome sequencing including 18 affected and 14 unaffected individuals. Sanger sequencing was performed according the standard protocol. The primer sequences were as follows: 5′-GATGGACCCCATTAGAGTAG-3′ and 5′-AGAGTAGTGACTCTACCCTC-3′. Sequence comparisons and analyses were performed using Chromas2 (Technelysium Pty Ltd., Tewantin, QLD, Australia).

### Histochemical staining and immunohistochemistry

The paraformaldehyde-fixed chondroma tissues from the HME patient were fixed with 1% paraformaldehyde (PFA; Sigma-Aldrich), rinsed and decalcified and embedded in paraffin. Paraffin-embedded chondroma tissues were sectioned (5-μm thick) and placed on glass slides. For histochemistry, the tissue slides of HME were dewaxed in xylene, hydrated with graded ethanol and stained by toluidine blue (TB) and hematoxylin and eosin (H&E), respectively.

For immunohistochemistry, the dewaxed and hydrated chondroma sections of HME were treated with 3% hydrogen peroxide solution for 10 min, rinsed with PBS followed by 5% donkey serum blocking and incubated with rabbit polyclonal anti-EXT1 antibody (1:50 working dilution; Abcam Plc, UK) at 4°C overnight. The chondroma sections were then incubated with the secondary antibody (Golden Bridge Biotechnology, Zhongshan, China) at 37°C for 30 min and stained using the DAB substrate kit (Vector Laboratories, Burlingame, CA, USA) and Meyer’s hematoxylin.

### EXT1 mutation spectrum

We downloaded the mutation data according to the variant type of substitution, deletion, insertion, inversion, insertion or deletion and translocation from the Multiple Osteochondroma Mutation Database. Subsequently, we summarized the mutations which were located in the exon regions and drew the mutation spectrum with Circos plot.

## Results

We observed that the tissue structure (Fig. 1C) of HME was represented with a cartilage cap, covered by fibrous perichondrium and merged into an underlying spongy bone. The stained chondroma sections with H&E and TB are presented in Fig. 1D.

We used Human All Exon V3 Agilent (50 M) to capture the exome of 3 affected (I9, II3 and II19) and 3 unaffected (I10, II2 and II26) individuals in the HME family (Fig. 1A). After performing parallel sequencing, ~120 million bases and 80 million reads per individual in average were generated. The mapping rates against the human being reference (hg19) of all individuals achieved 99%. Approximately 92% of the target regions were sequenced at least 10 times (depth ≥10x) in each individual. For every individual, ~80,000 variants were identified within the captured regions.

We focused on those variants that were located in exon, UTR and splicing sites. Taking the familial dominant model of inheritance into account, the variants that appeared in all 3 affected individuals but not in any unaffected individual were selected. We then filtered against the available public databases (dbSNP). Furthermore, we only focused on nonsynonymous, stopgain, stoplose and frameshift variants. After all the previous filtrations, only 3 heterozygous variants, ATXN3, TRPM3 and C11orf40, remained and only one heterozygous deletion (c.1325delC) in EXT1 was probably damaging, as the EXT family has been reported to be associated with HME. No reports were found at this position in the Multiple Osteochondroma Mutation Database. Thus, we selected it for further study.

**Figure 2:**
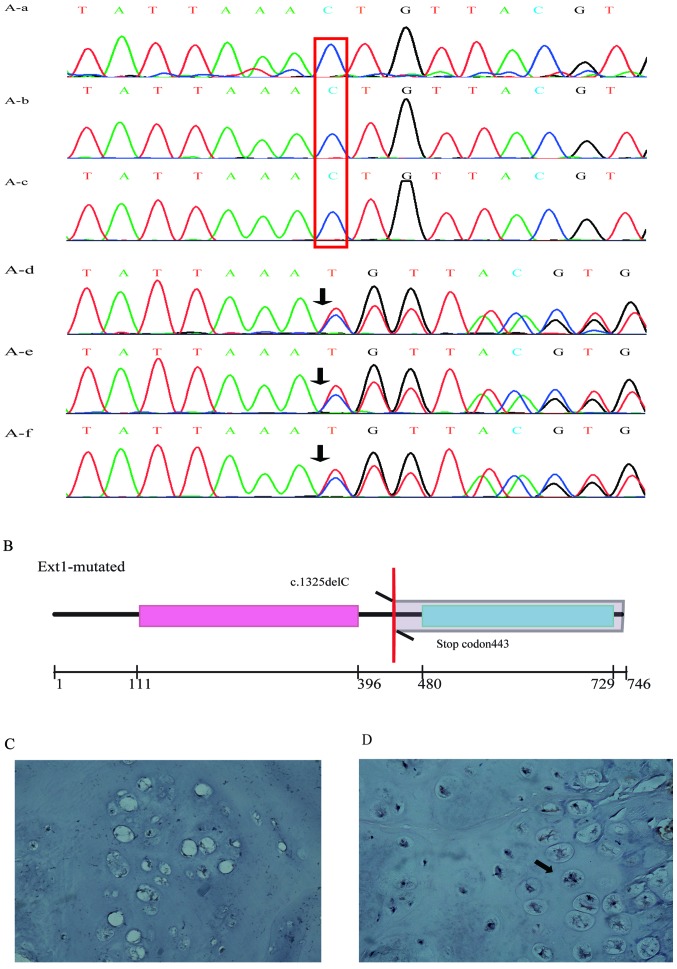
A new frameshift mutation and immunohistochemistry screening (20x). (Aa-c) Normal individuals (I10, II2 and II26). (Ad-f). Affected individuals (I9, II3 and II19). The red box indicates the normal type, the black arrows represent the mutation point. (B) Coding region structure of the exostosin-1 (EXT1) gene. Scale plate labels the 746-amino acid sequencing. The grey region indicates the truncation region of EXT1 proteins encoded by mutated EXT1 genes. (C and D) Immunohistochemical staining of EXT1 in chondrocytes in the superficial layers of cartilage caps of the proband and another hereditary multiple exostosis (HME) patient without EXT1 mutation from another family. The arrow represents a chondrocyte with functional EXT1.

To eliminate false-positive mutations, we examined and validated the mutations by Sanger sequencing in the source individuals. The heterozygous deletion g.chr8: 118834796 delC (Fig. 2A) was confirmed to be shared by all of the 3 studied patients but was absent in the normal individuals. The mutation led to a frameshift at codon 442 resulting in a change of serine to isoleucine and generated a stop codon at codon 443 causing the glycosyltransferase domain loss (Fig. 2B).

To further verify that the deletion was the disease-causing gene mutation in the HME family, we performed PCR and Sanger sequencing on all the family members and another 3 individuals (I1, I2 and II7) from another unrelated family (Fig. 1B). The mutation was confirmed to be shared by all the affected individuals except patient II22 and was absent in all of the healthy family members.

Immunohistochemistry was used to detect the protein expression level of EXT1 in these 2 unrelated families. We found that the EXT1 protein level of a HME patient (Fig. 2C) with the novel mutation was less than the level in an HME patient without the EXT1 mutation from another unrelated family (Fig. 2D).

**Figure 3:**
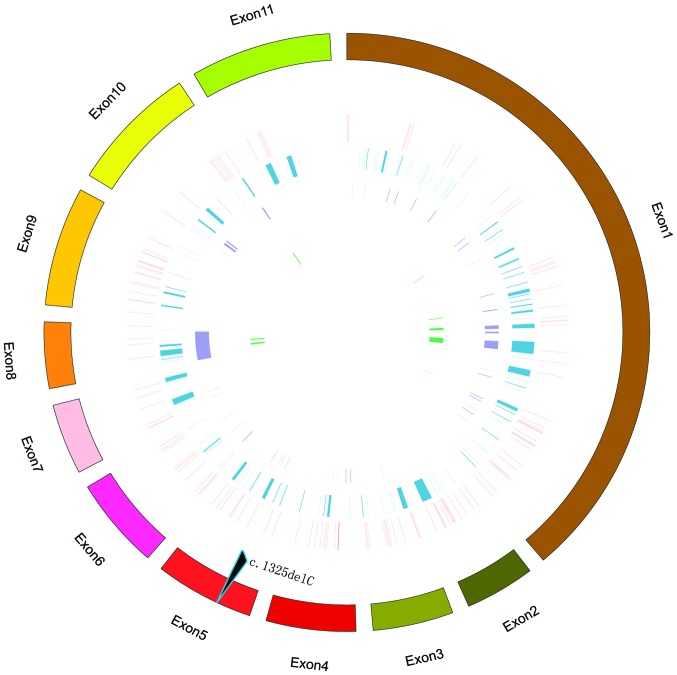
Mutation distribution of the entire exostosin-1 (EXT1) gene. The red rectangles represent single-base substitution mutations, the blue rectangles represent deletion mutations, the purple rectangles represent insertion mutations, the green rectangles represent insertion/deletion mutations. The black arrow indicates the deletion in the present study.

To further analyze the mutations in EXT1, we gathered all of the mutations in its 11 exons from the Multiple Osteochondroma Mutation Database. No inversion and translocation were found on all of the 11 exons. Substitutions and deletions were evenly distributed on the 11 exons, while small insertions were rarely locate in exon 2, 9 and 11 (Fig. 3).

## Discussion

The gene EXT1 locates on 8q24.1 and consists of 11 exons. EXT1 encodes a protein which possesses the activity of glucuronic acid sugar-based transfer enzyme and N acetyl glucosamine glycosyltransferase (4) which catalyzes the polymerization of HS chains at the endoplasmic reticulum and Golgi apparatus (18). HS is widely expressed on the cell surface, and as the component of extracellular matrix glycoprotein it mediates cell adhesion, signal transduction and the receptor ligand binding process. Previous studies have indicated that HS is an essential molecule and dysfunction could cause HME (19,20).

Herein, we found a novel c.1325delC mutation located in exons 5. This frameshift mutation was validated to be shared by all of the affected individuals except patient II22 and was absent in the healthy family members. Additionally, the mutation was not discovered in individuals from another HME family. As this damaging deletion mutation was not present in patient II22, further study will be carried out.

The c.1325delC mutation locates between the exostosin and glycosyltransferase domains. It led to a frameshift at codon 442 and resulted in a change of a serine to an isoleucine. The promptly premature stop codon at codon 443 caused loss of the glycosyltransferase domain, and the decrease in EXT1 protein expression was confirmed by immunohistochemisty. Because of the truncation, the EXT1 products may be unable to fold correctly and would rapidly degrade (21). This result is consistent with the reduced EXT1 protein observed in HME patients in previous studies (22,23). According to the statistics of all mutations on EXT1 in the Multiple Osteochondroma Mutation Database (24), all variant types were nearly equally distributed on the 11 exons.

Taken together, we report a novel frameshift mutation c.1325delC in the gene EXT1 by whole-exome sequencing. The mutation was validated by Sanger sequencing and its negative influence on the expression of EXT1 was examined via immunohistochemistry. Our results provide a new mutation in EXT1, further emphasizing the dysfunction of the EXT gene family as a cause of HME. This finding may be helpful for the early diagnosis and prenatal genetic screening of HME.

## Figures and Tables

**Table I:** Clinical characteristics of the subjects for exome sequencing.

Sample no.	Position	Clinical diagnosis	Blood	Age (years)	Gender	Analyzed by WES
13JX00006WB3	I10	/	Yes	65	Male	Yes
13JX00007WB2	I9	HME	Yes	62	Female	Yes
13JX000011LC2	II3	HME	Yes	27	Male	Yes
13JX00002LC2	II2	/	Yes	29	Male	Yes
13JX000017WB2	II19	HME	Yes	19	Male	Yes
13JX000019WB2	II26	/	Yes	39	Female	Yes

‘HME’, affected members; ‘/’, unaffected members; HME, hereditary multiple exostosis.
